# Quality-by-Design-Based Development of a Voxelotor Self-Nanoemulsifying Drug-Delivery System with Improved Biopharmaceutical Attributes

**DOI:** 10.3390/pharmaceutics13091388

**Published:** 2021-09-02

**Authors:** Aristote B. Buya, Romano Terrasi, Jérémie K. Mbinze, Giulio G. Muccioli, Ana Beloqui, Patrick B. Memvanga, Véronique Préat

**Affiliations:** 1 Advanced Drug Delivery and Biomaterials Group, Louvain Drug Research Institute, Université Catholique de Louvain, Avenue Mounier 73, B1.73.12, 1200 Brussels, Belgium; aristote.buya@uclouvain.be (A.B.B.); ana.beloqui@uclouvain.be (A.B.); 2 Pharmaceutics and Phytopharmaceutical Drug Development Research Group, Faculty of Pharmaceutical Sciences, University of Kinshasa, Kinshasa XI BP 212, Democratic Republic of the Congo; jeremie.mbinze@unikin.ac.cd (J.K.M.); patrick.memvanga@unikin.ac.cd (P.B.M.); 3 Bioanalysis and Pharmacology of Bioactive Lipids Research Group, Louvain Drug Research Institute, Université Catholique de Louvain, Avenue Mounier 73, B1.72.01, 1200 Brussels, Belgium; romano.terrasi@uclouvain.be (R.T.); giulio.muccioli@uclouvain.be (G.G.M.)

**Keywords:** SNEDDSs, bioavailability, quality-by-design

## Abstract

Low aqueous solubility and poor oral bioavailability are limiting factors in the oral delivery of voxelotor, an antisickling agent. To overcome these limitations, a voxelotor self-nanoemulsifying drug delivery system was developed. Various oils, surfactants, and cosurfactants were screened for their solubilization potential for the drug. The area of nanoemulsification was identified using a ternary phase diagram. An experimental mixture design and a desirability function were applied to select SNEDDSs that contain a maximum amount of lipids and a minimum amount of surfactant, and that possess optimal emulsification properties (i.e., droplet sizes, polydispersity index (PDI), emulsification time, and transmittance percentage). The optimized SNEDDS formulation was evaluated for the self-emulsifying time (32 s), droplet size (35 nm), and zeta potential (−8 mV). In vitro dissolution studies indicated a 3.1-fold improvement in drug solubility from the optimized SNEDDS over pure drug powder. After 60 min of in vitro lipolysis, 88% of the voxelotor loaded in the SNEDDS remained in the aqueous phase. Cytotoxicity evaluation, using Caco-2 cells, indicated the safety of the formulation at 0.9 mg/mL. The transport of the voxelotor SNEDDS across Caco-2 monolayers was significantly enhanced compared to that of the free drug. Compared to the drug suspension, the developed SNEDDS enhanced the oral bioavailability (1.7-fold) of voxelotor in rats. The results suggest that further development of SNEDDSs for the oral delivery of voxelotor is needed.

## 1. Introduction

During recent decades, the number of sickle cell disease (SCD) patients has increased significantly, making it the most common genetic disorder affecting millions of people worldwide, particularly in sub-Saharan Africa [1]. Several strategies have been applied in the treatment of this pathology, characterized by red blood cell sickling, vaso-occlusion, haemolytic anaemia, and vasculopathy leading to progressive organ damage [2]. The cellular sickling process can be reduced by increasing the oxygen affinity of haemoglobin S (HbS). The literature has reported that increasing the concentration of oxygenated HbS, without compromising oxygen delivery, is a promising approach to prevent red blood cell sickling and, subsequently, achieve long-term disease improvement [3].

Voxelotor (Vox), also known as GBT-440, is a small compound that binds to HbS and increases its affinity for oxygen. Vox improves the in vitro red blood cell flexibility and survival [4]. In a rat model, Vox prevented ex vivo red cell blood sickling [3]. The first-in-human studies in healthy volunteers and SCD patients showed that the tolerability and safety of Vox was associated with an increase in HbS oxygen affinity [5]. OXBRYTA^®^, a form of Vox, has been given accelerated approval by the U.S. Food and Drug Administration for patients aged 12 years and older [6]. However, the oral delivery of Vox has been thwarted because of its lipophilicity (log 3.54) and poor aqueous solubility (43 µg/mL) [5]. This aqueous solubility not only provides low oral bioavailability (36%), but also leads to considerable subject dose variability [5]. Furthermore, the absorption of Vox from the GI tract is affected by food intake [6]. When OXBRYTA^®^ was orally given to healthy human volunteers with a high-fat meal, it showed a 42- and 45-fold enhancement in AUC and C_max_, respectively, compared to the fasted state [6].

Lipid-based formulations have emerged as a promising strategy to improve the aqueous solubility and oral absorption of lipophilic drugs, and to decrease undesirable food effects [7]. The development of self-nanoemulsifying drug delivery systems (SNEDDSs) has provided one approach that is commonly adopted in this direction. SNEDDSs are anhydrous mixtures of oils, surfactants, and cosurfactants, that spontaneously form oil-in-water nanoemulsions with droplet sizes of less than 200 nm when exposed to GI fluids [8]. Compared to other lipid nanocarriers, SNEDDSs are easy to scale up and contain biodegradable excipients [9,10]. The food-associated effects of several lipophilic drugs, such as cinnarizine [11], torcetrapib [12], and itraconazole [13], have been nullified when encapsulated into SNEDDSs. Many SNEDDS formulations have been developed and optimized by taking into consideration the resulting emulsion droplet sizes after aqueous dispersion, through the use of empirical “trial and error” ternary diagram approaches, which consist of varying one factor at a time [14]. Unfortunately, such approaches are highly time consuming and require a number of experiments and resources. Furthermore, they often provide inadequate data to determine the impact of excipients on the performance of the formulation [10,15].

The use of the quality-by-design (Qbd) approach, applying the statistical design of experiments (DoE) for the systematic optimization of SNEDDSs has been reported to reduce expenditure in terms of time, resources, and developmental efforts. The Qbd approach provides an optimal amount of data and process understanding from a limited number of experiments [16]. A DoE applied during the component screening can provide more insight into excipient effects and interactions in the SNEDDS performance [17,18]. The Qbd approach has been used in the optimization of a wide variety of lipid-based formulations, including itraconazole microemulsions [19], rivaroxaban self-nanoemulsifying drug delivery systems [20], doxorubicin and curcumin coloaded liposomes [21], and rosuvastatin calcium solid lipid nanoparticles [22].

Therefore, the present work aims to use the Qbd approach for the development and optimization of Vox-loaded SNEDDSs. An experimental mixture design and a desirability function were applied to select SNEDDSs that contain a maximum amount of lipids and a minimum amount of surfactant, and that possess optimal emulsification properties (i.e., droplet sizes, polydispersity index (PDI), emulsification time, and transmittance percentage). Further, this work endeavours to evaluate the biopharmaceutical performance of the optimized Vox-SNEDDS in terms of in vitro dissolution, lipolysis, cytotoxicity, transport studies, and in vivo pharmacokinetic studies.

## 2. Materials and Methods

### 2.1. Materials

Voxelotor with a purity greater than 98% was purchased from MedChemExpress (Monmouth, NJ, USA). Cremophor-EL^®^ (polyoxyl-35 castor oil) was kindly provided by BASF (Ludwigshafen, Germany). Labrasol AFL^®^ (caprylocaproyl polyoxyl-8- glycerides), Transcutol HP^®^ (diethylene glycol monoethyl), Labrafil M^®^ 1944 CS (oleoyl polyoxyl-6-glycerides), Labrafil M^®^ 2125 CS (linoleoyl polyoxyl -6-glycerides), Capryol PGMC^®^ (propylene glycol monocaprylate type I), Lauroglycol^®^ 90 (propylene glycol monolaurate), and Maisine^®^ 35-1 (glycerol monolinoleate) were kind gifts from Gattefossé (Saint-Priest, France). Tween^®^ 80 (polysorbate 80), L-α-phosphatidylcholine (TLC), sodium taurodeoxycholate (NaTDC), 4-bromophenylboronic acid, porcine pancreatin extract (P7545, 8 × USP specification activity), and thiazolyl blue tetrazolium bromide (MTT) were all purchased from Sigma-Aldrich (St. Louis, MO, USA). Empty gelatine capsule shells (size “0”) were purchased from Capsugel Inc. (Morristown, NJ, USA). Formic acid, acetonitrile, methanol, and dimethylsulfoxide (DMSO) (all HPLC grade) were purchased from VWR (Copenhagen, Denmark). Purified water was used in all experiments. All other reagents were of analytical grade and used as received.

### 2.2. Analytical Methods

#### 2.2.1. HPLC–UV Method

An HPLC-UV system was used to quantify voxelotor. The HPLC (Shimadzu C 204353, Kyoto, Japan) was equipped with of an LC-20A pump, an SIL-20A autosampler and SPD-20A intelligent UV/VIS detector. A CC 250-4.6 Nucleosil 100-5, C18 HD HPLC column (Macherey-Nagel, Düren, Germany) was used for chromatographic separation. The mobile phase consisted of 20% *v*/*v* (water + 0.1% formic acid) and 80% *v*/*v* (acetonitrile + 0.1% formic acid) under isocratic mode. The velocity of the flow, sample load and wavelength of the UV detector were set at 1.0 mL/min, 10 µL and 272 nm, respectively. The HPLC–UV method was validated according to the current international regulatory guidelines [23]. In particular, the linearity, accuracy, precision, reproducibility, and repeatability of the method were assessed and are presented in Supplementary Table S1 and Supplementary Figures S1 and S2. The limit of quantification and of detection were 0.7 µg/mL and 0.2 µg/mL, respectively. The retention time of Vox was about 4.8 ± 0.15 min. 

#### 2.2.2. LC–MS Method

Voxelotor was extracted from rat serum samples (50 µL) in the presence of F21450908, which was used as an internal standard (30 pmol), by adding acetonitrile (400 µL) and hydrochloric acid (10 µL, 2 N). After an overnight incubation (−20 °C), the samples were centrifuged, and the supernatant was transferred to an injection vial. The samples were analysed using a Waters Xevo TQ-S tandem quadrupole mass spectrometer, coupled to an Acquity UPLC class H system (Waters, Milford, MA, USA). A Kinetex LC-18 (150 × 4.6 mm, 5 µm) column (Phenomenex) and a 10-min gradient between MeOH-water (75:25, *v*/*v*) (with 0.1% acetic acid) and MeOH (with 0.1% acetic acid) were used. Ionization (positive mode) was obtained using an ESI probe. The quantification transitions for voxelotor and F21450908 were 338.1 → 200.0 and 342.1 → 222.1, respectively. The ratio between the area under the curve (AUC) of voxelotor and of the internal standard was reported using a calibration curve (obtained under identical conditions). To establish the LOD and LOQ, plasma (50 µL) was spiked with voxelotor at several levels and analysed using the same protocol. The values were 337 × 10^−5^ and 1126 × 10^−5^ µg/mL, respectively.

### 2.3. Optimization of the SNEDSS Formulation

#### 2.3.1. Equilibrium Solubility of Vox

The solubility of Vox was studied in the selected excipients (oils, surfactants and cosurfactants). An excess amount of Vox was added to 500 mg of each excipient under stirring (100 rpm, 37 °C) in a shaking incubator (Infors AG, Bottmingen, Switzerland) for 48 h. The resultant samples were centrifuged at 4000× *g* for 15 min (37 °C) using an Eppendorf centrifuge 5804 R (Hamburg, Germany). The supernatant was diluted with acetonitrile, and the concentration of Vox was determined by HPLC-UV.

#### 2.3.2. Screening of Surfactants and Cosurfactants for Self-Emulsifying Ability

The self-emulsification capacity of the surfactants was studied as described by Date et al. [24], with minor modifications. In brief, mixture of the selected oil and surfactant at a ratio 1: 1 (*w*/*w*) was heated (40–45 °C) under gentle stirring. The resulting mixture (500 mg) was dispersed in 10 ml of deionized water under gentle stirring. Visual observation was carried out to assess the relative turbidity. The resulting dispersions were allowed to stand for 2 h and the transmittance percentage values were determined at 550 nm using a NanoDrop^TM^ 2000 spectrophotometer (Thermo Fisher Scientific Inc., Waltham, MA, USA) with deionized water as a control.

To assess the emulsification ability of the cosurfactants and cosolvents, each of them was mixed with the selected surfactant at a 2:1 (*w*/*w*) ratio. The selected oil was added to this mixture at a 1:3 ratio under stirring and heat (40–45 °C). The resulting dispersions were analyzed as mentioned for the surfactant screening.

#### 2.3.3. Development of SNEDDSs Employing “Qbd”

##### Ternary Phase Diagram

A ternary phase diagram of the oil, surfactant, and cosurfactant was plotted, with each representing an apex on the triangle. Forty ternary mixtures (with varying compositions of oil, surfactant, and cosurfactant) chosen from the solubility studies were prepared. The mixture (500 mg) was accurately weighed and dispersed in 200 mL of deionized water (37 °C) under gentle agitation (50 rpm). Visual observation was carried out immediately to investigate the occurrence of self-nanoemulsification. Dispersions with measured particle sizes of less than 200 nm were used to draw the nanoemulsion area of the diagram [25]. Phase diagrams were constructed using Chemix School version 3.60 software (Arne Standnes, Bergen, Norway).

##### Preparation of SNEDDSs for the Experimental Design

The SNEDDSs were prepared by mixing (100 rpm, 20 ± 5 °C) the oil, surfactant, and cosurfactant at predetermined amounts, as per the design (Table 1). The final mixtures were stirred for dissolution until clear preparations were obtained and were then stored (20 ± 5 °C) for further studies.

##### Experimental Design

A 16-run custom design, using the Bayesian D-optimality quality criterion, was generated to estimate a full cubic model for the three critical formulation variables. The design was blocked on 4 days of 4 runs each. The experimental design and statistical analysis were executed using JMP Pro^®^ 14.3.0 (SAS Institute, New York, NY, USA). The independent variables and their respective levels were selected based on solubility and ternary phase diagram studies. The proportions of oil (X_1_, % *w*/*w*), surfactant (X_2_, % *w*/*w*), and cosurfactant (X_3_, % *w*/*w*) were considered independent variables (factors), whereas the dependent variables (responses) were emulsification time (Y_1_, s), droplet size (Y_2_, nm), PDI (Y_3_), and transmittance percentage (Y_4_, %). The amount of the components was held constant (1 g), while the ratio of the three was varied. Sixteen SNEDDS formulations were prepared and are presented in Table 1. The data obtained from the response measurements were analysed using a mixed model, with the day as a random variable, and a fixed full model on our explanatory variables. When possible, the model was simplified, taking into account the linear constraints between the factors, to raise the model power. The correlation of factors with response variables was then fitted into different mathematical models (quadratic, cubic, or special cubic). The model quality was estimated using the R-squared, adjusted R-squared, root mean square error, and *p*-value of the F-test associated with the contribution of the variables in the model (critical *p*-value = 5%). The models were reduced by removing nonsignificant higher-degree terms to make them cubic, then quadratic, and, finally, first-order. Next, a desirability function using JMP Pro^®^ 14.3.0 was applied to optimize factors for desirable responses.

#### 2.3.4. Evaluation of Dependent Variables

##### Emulsification Time (Y1)

Each SNEDDS formulation (1 g) was dispersed in 250 mL of deionized water under gentle stirring (100 rpm, 37 ± 0.5 °C) [14]. The emulsification time was recorded as time in seconds required to obtain a clear dispersion [26].

##### Droplet Size and PDI (Y2 and Y3)

The droplet size and PDI were determined by dynamic light scattering (DLS) at 37 °C using a Nano ZS system (Malvern Instruments, Malvern, UK) with a water dispersant refractive index of 1.330. One gramm of the formulations were dispersed in 250 mL [14] of filtered deionized water and allowed to stand for 1h prior the analysis. The zeta potentials were determined via electrophoretic mobility using the same instrument. All measurements were done in triplicate using disposable polystyrene cuvettes (Malvern Instruments, UK). 

##### Transmittance Percentage (Y4)

One gram of the formulations were emulsified in 250 mL of deionized water and allowed to stand for 1h The transmittance percentage of resulting dispersions were mesuered at 550 nm [27,28] using a UV-visible spectrophotometer (Thermo Fisher Scientific Inc.) wiht deionized water as a control.

#### 2.3.5. Transmission Electron Microscopy (TEM)

The morphology of the optimized nanoemulsion droplet was examined using a transmission electron microscope (Tecnai 10 microscope, FEI, Hillsboro, OR, USA) with a 100 kV accelerating voltage. A 0.5-mL droplet of the reconstituted SNEDDS formulation was positioned on carbon-coated 300 mesh grids, followed by negative staining with a 0.2% aqueous solution of uranyl acetate.

### 2.4. In Vitro Characterization of Vox-Loaded SNEDDS

#### 2.4.1. In Vitro Dissolution Studies

Dissolution studies were carried out using a USP Dissolution Tester (Apparatus II, Model Sotax AT7, CH-4008, Basel, Switzerland) with 500 mL hydrochloric acid USP buffer (pH 1.2), phosphate buffer (pH 6.8) [29], and biorelevant medium (FeSSGF and FeSSIF). The speed of the paddle and the temperature were adjusted to 100 rpm and 37 ± 0.5 °C, respectively. The FeSSGF (fed state simulated gastric fluid) and FeSSIF (fed state simulated intestinal fluid) were prepared as per the method reported by Jantratid and Dressman [30]. Hard gelatine capsules (size “0”) were filled with 50 mg of pure Vox or 600 mg of Vox-loaded SNEDDS (equivalent to 50 mg of Vox), and placed in the dissolution tester. At predetermined time intervals, an aliquot (2 mL) was withdrawn and replenished with an equivalent volume of fresh and preheated (37 °C) medium. The withdrawn samples were centrifuged (4000× *g*) for 10 min and filtered through 0.22-µm Rotilabo^®^ syringe filters (Carl Roth, Karlsruhe, Germany). Appropriate dilutions in acetonitrile were performed prior to quantitative HPLC–UV analysis.

#### 2.4.2. In Vitro Lipolysis

In vitro lipolysis study was performed as described previously [31], with minor modifications. The equipment consisted of a compact stirrer (Mettler Toledo, Greifensee, Switzerland), an IKA C-MAG HS7 thermostat-jacketed glass reaction vessel (Staufen, Germany), a T5 Mettler Toledo pH-stat titration unit (Greifensee, Switzerland) containing a combined pH Ag/AgCl electrode (DGI 115-SC) and a 30-mL DV 1020 Mettler Toledo autoburette (Greifensee, Switzerland).

One gram of Vox-SNEDDS formulation was dispersed in 40 mL of lipolysis buffer (containing 1.4 mM CaCl_2_.2H_2_O, 0.75 mM TLC, 2 mM Tris-maleate, 3 mM NaTDC and 150 mM NaCl) for 20 min. Afterward, the pH was automatically set to 6.5, and in vitro lipolysis was started by adding 4 mL of pancreatin extract containing lipase (lipase activity equivalent to 8X USP specifications) and other pancreatic enzymes (ribonuclease, protease and amylase). The enzyme extract was prepared before each experiment by mixing 5 mL of lipolysis buffer with 1 g of pancreatic powder and 20 µL of NaOH solution (0.5 M) to reach the desired pH (6.5). The resulting enzyme dispersion was centrifuged (4000× *g*) for 15 min.

The fatty acids released during in vitro lipolysis were automatically titrated with 0.05 M NaOH to maintain the pH at 6.5. Lipolysis medium (2 mL) was withdrawn in 5-min intervals up to 1h of the experiment, and 10 µL of 1.0 M 4-bromophenylboronic acid (in methanol) was added to inhibit the enzyme activity. This process was followed by ultracentrifugation (6700× *g*, 4 °C MiniSpin, Eppendorf AG, Hamburg, Germany) for 20 min, resulting in the separation of the digestion content in a off-white pellet and clear supernatant. The supernatant was collected and Vox concentration was determined by HPLC-UV.

#### 2.4.3. X-ray Powder Diffraction (PXRD)

To elucidate the solid state of the precipitated Vox during in vitro lipolysis, the pellets retrieved at the end of the experiment were analysed by X-ray powder diffraction. An X-ray diffractometer (PXRD, Stoe Stadi P, Darmstadt, Germany), with CuKα as the radiation source (1.542 Å), was used. The radiation voltage and amperes were set to 40 kV and 40 mA, respectively. All PXRD profiles were obtained at room temperature in the angular range of 2θ = 5–60°, at a speed of 0.04° per second.

#### 2.4.4. Stability of the Vox-SNEDDS Formulation

SNEDDS formulations were stored for 6 months at room temperature and evaluated for optical clarity, droplet size, zeta potential, emulsification time, and drug content.

### 2.5. In Vitro Cell Line Study

The in vitro experiments were performed with Caco-2 cells. The cells were cultured in medium containing Dulbecco’s modified Eagle’s minimal essential medium (DMEM) supplemented with 1% (*v*/*v*) L-glutamine, 10% (*v*/*v*) heat-inactivated foetal bovine serum (HyClone^®^, Thermo Fisher Scientific Inc.), penicillin/streptomycin solution (10 units/10 µg/mL) and 1% (*v*/*v*) nonessential amino acids. The cells incubation was done in a humidified atmosphere (37 °C) containing 10% CO_2_. The cells were subcultured weekly once they reach 80% confluence.

#### 2.5.1. Cell Viability Assay

The cell viability against the optimized formulation was assessed as described by Memvanga et al. [32]. In brief, Caco-2 cells were seeded on 96-well culture plates (2 × 10^4^ cells/well; 100 µL per well) and incubated in the culture media. After 24 h, the cells were washed with phosphate-buffered saline (37 °C) and treated with 100 µL of unloaded-SNEDDS or free Vox at various concentrations (from 0.3 to 4 mg/mL) diluted with Hank’s salt balanced solution (HBSS). After 2h of incubation, the cell were washed and treated with 100 µL of MTT solution (0.5 mg/mL in DMEM) and were incubated for 3 h (37 °C). To solubilize the formazan crystals formed during the incubation, 200 µL DMSO was added and the product of reaction was measured at 545 nm using a Multiskan Spectrum microplate reader (Thermo Fisher Scientific Inc.). The cell viability of the control cells (treated with HBSS) was defined as 100%. 

The cell viability rates of the samples were calculated according to the following equation:

Cell viability (%) = A_s_/A_c_ × 100 (1)

where A_s_ is the sample absorbance, and A_c_ is the absorbance measured after treating the cells with HBSS.

#### 2.5.2. Cell Culture for Transport Studies

The in vitro transport experiments were carried out as described by Memvanga et al. [32]. Caco-2 cells (5 × 10^5^ cells/well) were seeded on 12-well cell culture inserts with a 0.9 cm^2^ surface area (Corning Costar^®^, NY, USA) and 1-µm pore diameter. Culture medium was replaced every two days and was added to the apical (0.5 mL) and basolateral (1.2 mL). The cells were incubated for 21 days to allow the differeciation until the measured transepithelial electrical resistance (TEER) increased to 400 ohm/cm^2^. The TEER was measured using a voltmeter with a chopstick electrode (World Precision Instrument, Sarasota, USA). Thirty minutes before the experiments, the cells were incubated with HBSS (37 °C), and the TEER values of the monolayers were mesured in triplicate. Apical to basolateral (AB) transport experiments across Caco-2 cell monolayers were conducted by adding 0.5 mL of Vox suspension (0.9 mg/mL Vox in HBSS) or 0.5 mL of dispersed Vox-SNEDDS in HBSS (0.9 mg/mL Vox-SNEDDS, i.e., 75 µg/mL Vox) on the apical side of the inserts, and 1.2 mL HBSS on the basolateral side. For the basolateral to apical transport experiments (BA), 1.2 mL of Vox suspension (0.9 mg/mL Vox in HBSS), or 1.2 mL of dispersed Vox-SNEDDS in HBSS (0.9 mg/mL Vox-SNEDDS, i.e., 75 µg/mL Vox), was added to the basolateral side, while the apical side was filled with 0.5 mL HBSS. 

After 2 h, TEER values of monolayers were determined in triplicate, and Vox content in acceptor compartments (basolateral for AB or apical for BA) was determined after appropriate dilutions by HPLC–UV. The apparent permeability coefficient (P_app_) was determined using the following equation:

(2) $$P_{\mathrm{app}}=\frac{dQ}{dt}\times \frac{1}{CoA}$$

where *dQ*/*dt* (transport rate) is the amount of Vox (μg) appearing per time unit (s) in the receiver compartment, *C_o_* is the initial concentration in the donor compartment (μg/mL), and *A* is the surface area of the insert (*A* = 0.9 cm^2^).

### 2.6. Pharmacokinetic Study

Male Sprague Dawley Fisher rats with a mean body weight of 300 g were obtained from Janvier Labs (Saint Berthevin, France). All rats were housed in a light-controlled room at a temperature of 20 ± 5 °C and a relative humidity of 25 ± 5%. All animal experiments were approved in March 2020 by, and performed in accordance with, the local animal committee (2020/UCL/MD/06, March 2020).

Before the experiments, the rats were divided into three groups (*n* = 18) and fasted for 12 h with free access to water. Group 1 animals were orally administered 1 mL of pure Vox suspension (in 0.5% sodium carboxyl methylcellulose) at a drug dose of 7.2 mg/kg, and group 2 animals were orally administered a Vox-loaded SNEDDS at a dose of 7.2 mg/kg. For intravenous administration, group 3 animals were administered 0.5 mL of Vox solution (in normal saline buffer containing 10% (*w*/*v*) Tween^®^ 80) via the tail vein at a dose of 1.6 mg/kg [3]. Blood samples (0.25 mL) were withdrawn from the tail vein using heparinized capillaries at 0.5, 1, 2, 6, 10, 24, 36, 48, and 72 h. The blood samples were centrifuged at 4000× *g* (10 min) to separate the plasma. The samples were stored at −80 °C until analysis by LC–MS. A noncompartmental pharmacokinetic analysis was used to determine the pharmacokinetic behaviour of voxelotor. The pharmacokinetic parameters were computed using the PK solver programme (Microsoft Excel) with the trapezoidal rule. Statistical analysis of in vivo pharmacokinetic data was conducted using a two-tailed unpaired Student’s *t*-test, with *p* values < 0.05 considered significant.

The absolute bioavailability (*F*) was calculated as follows:

(3) $$F\;\left(\%\right)=\frac{\mathrm{AUC}\;o/\mathrm{dose}\;o}{\mathrm{AUC}\;i/\mathrm{dose}\;i}\times 100$$

where AUCo and AUCi are the areas under the curve of the oral groups (o) (SNEDDS and suspension) and the intravenous group (i), respectively.

## 3. Results and Discussion

### 3.1. Excipient Screening

**Figure 1:**
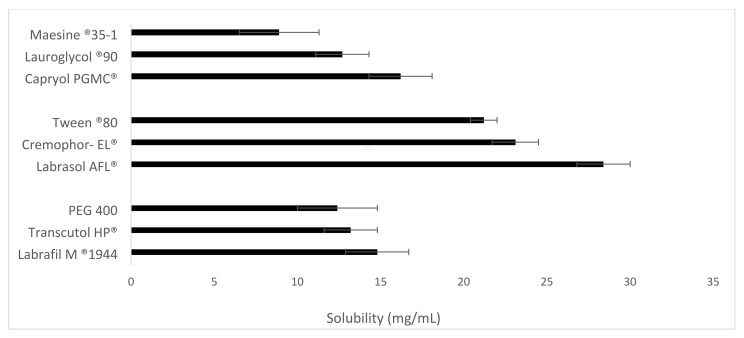
Solubility of Vox (mg/mL) in various oils, surfactants, and cosurfactants at 37 °C. Data are expressed as the mean ± SD, *n* = 3.

SNEDDS formulations are prepared to enhance the aqueous solubility and oral bioavailability of drugs. Excipient screening guides the right selection of components for this formulation. The choice of the excipients was based on the literature. The screening of oils, surfactants, and cosurfactants was performed, based on emulsification and solubility studies. As presented in Figure 1, among the oils tested, Vox showed the highest solubility in Capryol PGMC^®^ (16.6 ± 5.2 mg/mL). Therefore, Capryol PGMC^®^ was selected as the oily phase for further studies. The solubility of Vox in various surfactants is presented in Figure 1. Only hydrophilic surfactants (HLB > 12) were tested, as they favour the occurrence of oil-in-water emulsions [33,34]. Labrasol AFL^®^ yielded the highest solubility (37.4 ± 2.8 mg/mL), followed by Cremophor-EL^®^ (27.2 ± 0.7 mg/mL). However, the selection of surfactants was primarily based on their emulsification efficiency, rather than their ability to solubilize the drug [34]. Good solubility of the drug in the surfactant was considered an additional advantage regarding avoiding drug precipitation [35].

The transmittance percentage values of various oil-surfactant dispersions were measured (Table 1), and clearly distinguished the ability of Labrasol ALF^®^ and Cremophor-EL^®^ to emulsify Capryol PGMC^®^. Cremophor-EL^®^ exhibited a higher emulsification efficiency (99.3%), whereas Labrasol ALF^®^ showed a lower emulsification efficiency, as indicated by the lower transmittance percentage value (60.5%). Thus, Cremophor-EL^®^ was selected as the surfactant for further investigation.

Cosolvents and cosurfactants are used to cooperate with the surfactant to reduce the interfacial tension, increase the drug solubilization, and enhance the dispersibility, which resulted in improved emulsification and a reduced particle size. Among all the cosurfactants and cosolvents tested, Labrafil M^®^ 1944 CS was selected, owing to its higher drug solubilization value (Figure 1) and better emulsification efficiency (Supplementary Table S2).

### 3.2. Ternary Phase Diagram

**Figure 2:**
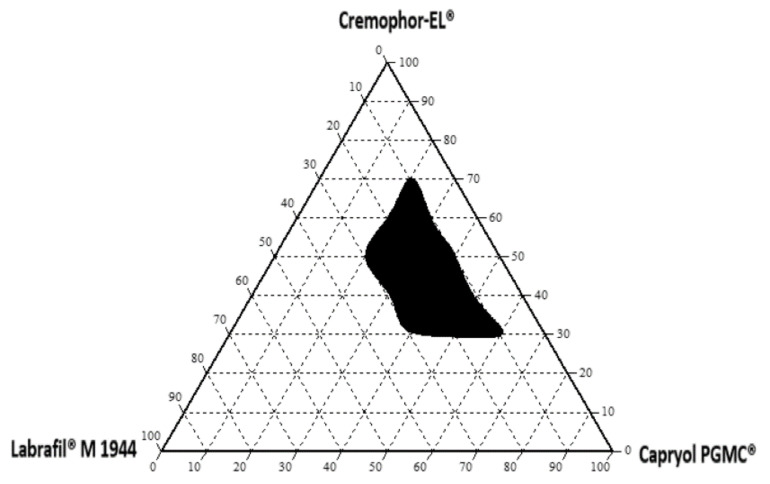
Phase diagram of oil (Capryol PGMC^®^), surfactant (Cremophor-EL^®^), and cosurfactant (Labrafil M^®^ 1944 CS).

Based on the emulsification and solubility studies, a ternary phase diagram was constructed in the absence of the drug to identify the self-nanoemulsifying region, as illustrated in Figure 2. The shaded area indicates the nanoemulsification region with a low droplet size (<200 nm). This study indicated that 20–60% (*w*/*w*) Capryol PGMC^®^ (oil), 30–70% (*w*/*w*) Cremophor-EL^®^ (surfactant), and 10–30% (*w*/*w*) Labrafil M^®^ 1944 CS (cosurfactant) ternary mixture (total 100%) showed a clear area that could be used to optimize the SNEDDS formulations using the D-optimal mixture design approach.

### 3.3. Statistical Analysis and Optimization of SNEDDS Formulations

According to the results obtained from the ternary phase diagram, ranges of factors were fixed as follows: 20% ≤ Capryol PGMC^®^ (X_1_) ≤ 60%, 30% ≤ Cremophor-EL^®^ (X_2_) ≤ 70%, and 10% ≤ Labrafil M^®^ 1944 CS (X_3_) ≤ 30%. The response variables were taken as the emulsification time (Y_1,_ s), droplet size (Y_2_, nm), PDI (Y_3_), and transmittance percentage (Y_4_, %), due to their impact on the SNEDDSs performance [16]. The 16 runs of the design and the measurements of the four responses are presented in Table 2. The statistical validity evaluation of the generated models (Table 3) confirmed that the mathematical models used for all the response variables were satisfactory and adequate (model *p* value > F < 0.05). Furthermore, model R-square and adjusted R-square values for each response variable indicated an excellent fit to the data.

#### 3.3.1. Self-Emulsification Time (Y_1_)

**Figure 3:**
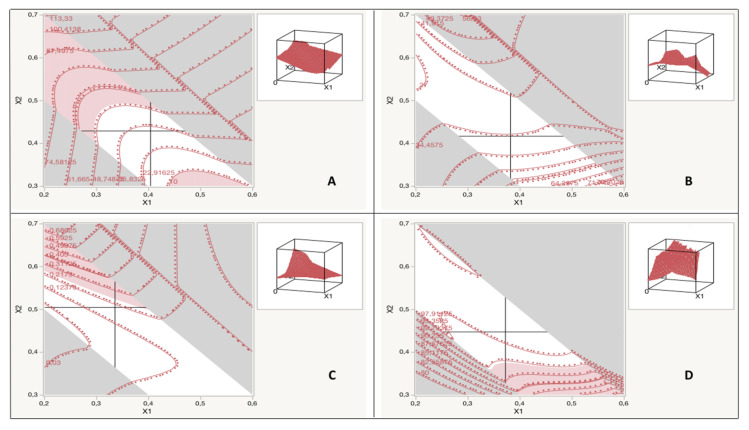
Contour profiler correlating independent variables with the (**A**) self-emulsification time (Y_1_), (**B**) droplet size (Y_2_), (**C**) PDI (Y_3_), and (**D**) transmittance percentage (Y_4_).

The self-emulsification ability of the SNEDDSs could effectively be estimated by determining the emulsification time. The correlation between the self-emulsification time and independent variables is presented in Table 4, Figure 3A, and Equation (4).

**Self-emulsification time (Y_1_, s)** = 11.9 X_1_ + 114.2 X_2_ + 144.9 X_3_ − 26.4 X_1_X_2_ − 230.6 X_1_X_3_ − 189.7 X_2_X_3_ r^2^ = 0.945; model = quadratic (4)

A significant positive effect (*p* < 0.05) was observed for the surfactant concentration (X_2_) on the time of emulsification. When the surfactant concentration rose, the emulsification time was found to increase. This phenomenon could be related to the high intrinsic viscosity of Cremophor-EL^®^ (600–750 mPas), reducing water penetration into the complex colloidal structure formed on the surface of the droplets [36]. In addition, as reported by Croy et al. [37], Cremophor-EL^®^ has a lower core polarity than other nonionic surfactants, which delays the penetration of water through the droplets during the emulsification process.

#### 3.3.2. Droplet Size (Y_2_) and PDI (Y_3_)

The size of the globule and its distribution are crucial in self-emulsification, as they determine the rate and extent of drug release [16]. The correlation between globule size, or PDI, and the independent variables is shown in Table 4, Figure 3B,C, and Equations (5) and (6).

**Droplet size (Y_2_, nm)** = 84.6 X_1_ + 46.4 X_2_ + 71.5 X_3_ − 125.6 X_1_X_2_ − 70.8 X_1_X_3_ − 117.3 X_2_X_3_ + 23.1 X_1_X_2_X_3_ − 114.3 X_1_X_2_(X_1_ − X_2_) + 6.8 X_1_X_3_(X_1_ − X_3_) − 46.1 X_2_X_3_(X_2_ − X_3_) r^2^ = 0.985; model = cubic (5)

**PDI (Y_3_)** = 0.1 X_1_ + 0.7 X_2_ + 0.0 X_3_ − 0.8 X_1_X_2_ + 0.2 X_1_X_3_ − 1.7 X_2_X_3_ r^2^ = 0.905; model = quadratic (6)

The results demonstrate that the droplet size, and its distribution, were significantly (*p* < 0.05) influenced by the oil and surfactant concentrations. Increasing the Capryol PGMC^®^ concentration up to, or above, 50% (*w*/*w*) induced a linear increase in the droplet size and PDI. This phenomenon could be attributed to an increase in hydrophobicity, owing to the lower amount of surfactant [38]. Conversely, there was a linear decrease in the globule size and PDI, with an increase in surfactant concentration from 30 to 60% (*w*/*w*); a phenomenon that could be attributed to the surface-tension-lowering property of the surfactant at the oil-water interface, which reduces the energy that is free for emulsification [39]. However, above a surfactant concentration of 60% (*w*/*w*), there was a remarkable increase in droplet size and PDI, which could be explained by one or both of the following reasons: (1) excess water penetration into the oil droplets, causing massive interfacial disruption and the ejection of highly polydispersed droplets; (2) a possible condensation phenomenon and the multilayer formation of additional surfactant into the droplets. In accordance with previous studies [28,40], increasing the amount of hydrophilic components above 60% (*w*/*w*) promoted an increase in the droplet size and PDI of the nanoemulsion formed upon the SNEDDSs’ dispersion in an aqueous environment. Furthermore, an antagonistic effect of the interaction between the oil and surfactant concentrations on droplet size and PDI was observed. Simultaneously increasing the oil and surfactant concentrations significantly reduced the nanoemulsion droplet size and PDI. In agreement with previous studies [17], medium chain monoglycerides, such as Capryol PGMC^®^, were likely to increase the interfacial fluidity of the surfactant boundaries in the micelles. The entrapment of Capryol PGMC^®^ in a high HLB surfactant (i.e., Cremophor-EL^®^) enhanced the emulsification process upon water dispersion, resulting in the narrow size distribution of the oil droplet. Accordingly, the oil and surfactant combination has a considerable impact on the droplet size, PDI, and the self-emulsification of the SNEDDSs upon dispersion in aqueous environments [34].

#### 3.3.3. Transmittance Percentage

The transmittance percentage is a useful tool to meet the optical clarity of the diluted SNEDDSs with water. The correlation between the transmittance percentage and independent variables is presented in Table 4, Figure 3D, and Equation (7).

**Transmittance percentage (Y_4_, %)** = 80.8 X_1_ + 97.9 X_2_ + 151.5 X_3_ + 43.2 X_1_X_2_ − 107.6 X_1_X_3_ − 102.2 X_2_X_3_ + 161.8 X_1_X_2_X_3_ + 49.3 X_1_X_2_(X_1_ − X_2_) + 74.9 X_1_X_3_(X_1_-X_3_) + 77.4 X_2_X_3_(X_2_ − X_3_) r^2^ = 0.985; model = cubic (7)

Initially, the transmittance percentage increased with an increasing oil amount (up to 40% *w*/*w*). However, the transmittance percentage decreased with a further increase in the oil concentration. This phenomenon might be attributed to an increase in globule size, owing to the decrease in the surfactant concentration, resulting in the coalescence of the oil globule [41]. Conversely, the increase in the surfactant content exhibited a significant positive effect on the transmittance percentage, which could be explained by the observation that more surfactant could sufficiently reduce the interfacial tension, stabilize the oil-water interface, and minimize the droplet size [35]. For the droplet size, a significant (*p* < 0.05) positive interaction (an increase in the transmittance percentage) was observed between the oil and surfactant concentrations. Based on this result, it could be concluded that the transmittance percentage correlated with the droplet size, although in the opposite direction (Y = 290 − 2.75X, r^2^ = 0.896, *p* < 0.05). As reported previously [42,43], the measurement of the transmittance percentage is a key parameter in a SNEDDSs’ characterization, and can serve as an alternative indicator of droplet size.

#### 3.3.4. Optimization by Desirability Function

**Figure 4:**
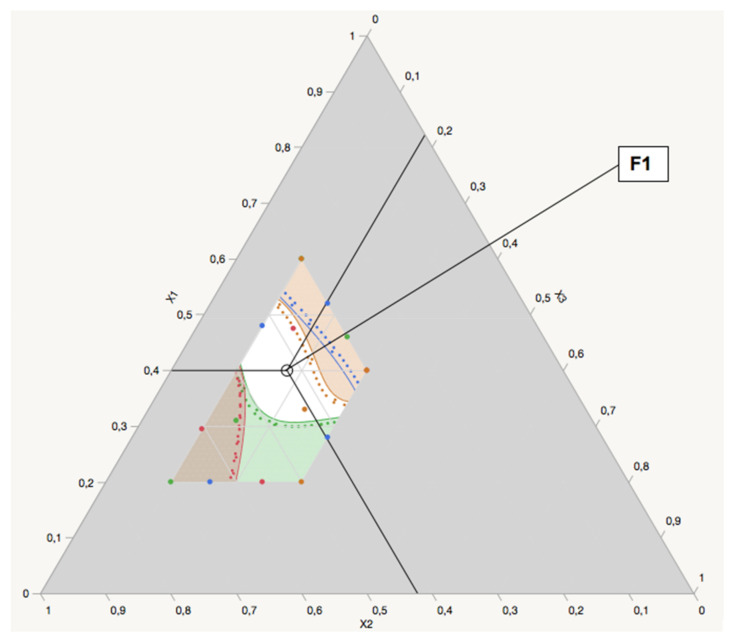
Mixture profiler of the isotropic blend prepared as per the D-optimal design. Prediction formula for the following: green, emulsification time (Y_1_); blue, droplet size (Y_2_); red, PDI (Y_3_), brown, transmittance percentage (Y_4_); white, optimum area.

Emulsification time, droplet size, PDI, and transmittance percentage are each commonly studied response variables for a SNEDDSs’ optimization [7,10,28]. A short emulsification (<50 s) contributes to the rapid release of the drug and a rapid onset of action [44]. A small particle size with a narrow distribution has a positive effect on the oral bioavailability of a drug encapsulated in a SNEDDS [45]. In addition, the generation of a smaller dispersion, after aqueous dilution or lipolysis, is generally necessary because it is known that the dose variability of these formulations can be minimized after oral ingestion [46,47]. The transmittance percentage is a useful tool for evaluating the isotropic properties of the resulting nanoemulsions. A high transmittance value (≥95) indicates clarity of the dispersion [10]. Thus, the targeted ranges of the responses were fixed as follows: Y_1_ (≤50 s), Y_2_ (≤100 nm), Y_3_ (≤0.25 PDI), and Y_4_ (≥95%) [16,27,48]. Under these conditions, the desirability function combines the four responses to determine an overall optimum region. Figure 4 shows the profiler desirability with the optimum region (in white). To maximize the drug loading capacity, only the SNEDDS formulations with a high oil content (>35%) were chosen for verification. The results suggest that a SNEDDS formulation comprised of Capryol PGMC^®^ (40% *w*/*w*), Cremophor-EL^®^ (43% *w*/*w*), and Labrafil M^®^ 1944 CS (17% *w*/*w*) fulfilled the requirements for an optimum formulation, and was chosen for verification. To validate the predictability of the generated mathematical models, the optimum formulation (F1) was prepared according to the above values of factors and subjected to the response measurements (i.e., emulsification time (s), globule size (nm), PDI, transmittance percentage (%), and zeta potential (mV)). As presented in Table 5, values obtained from checking F1 were not significantly different (bias less than 5%) from the predicted values. Thus, the validity of the generated model was established. F1 was considered an optimum formulation and was used for further in vitro and in vivo evaluation.

### 3.4. Transmission Electron Microscopy

**Figure 5:**
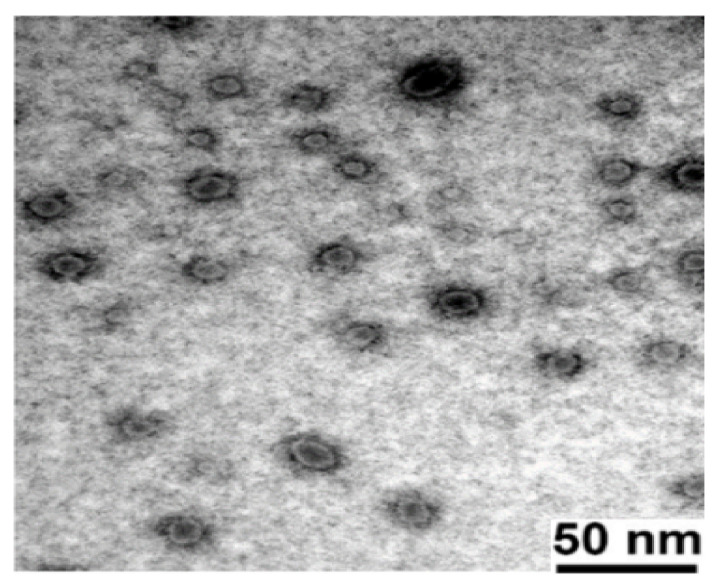
Transmission electron microphotography (TEM) of the reconstituted F1 formulation.

The morphology of the reconstituted F1 (as shown in Figure 5) was observed using TEM. The nanoemulsion droplets had spherical shapes and narrow size distributions.

### 3.5. In Vitro Dissolution Studies

**Figure 6:**
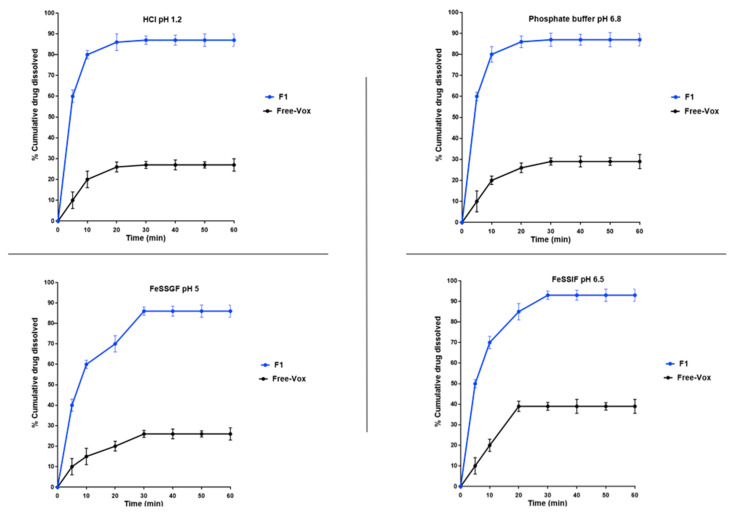
Dissolution profile of F1 and pure drug in various dissolution media using a paddle apparatus at 37 ± 0.5 °C. Data are expressed as the mean ± SD, *n* = 3. FeSSGF—fed state simulated gastric fluid; FeSSIF—fed state simulated intestinal fluid.

The in vitro dissolution of Vox in F1 was evaluated and compared to that of the pure drug in different dissolution media (pH 1.2 HCl, pH 6.8 phosphate buffer, FeSSGF and FeSSIF) (Figure 6). Compared to F1 (87%, 86.6 µg/mL), the pure drug showed relatively low dissolution in pH 1.2 HCl and pH 6.8 buffer, with approximately 28% (27.6 µg/mL) and 29% (28.8 µg/mL) of the dose being dissolved, respectively. The higher drug dissolution from the formulation could be attributed to the reduction in particle size and the increase in surface area and drug solubility [49]. The dissolution of Vox was enhanced in the simulated intestinal media (FeSSIF). Approximately 38% (37.7 µg/mL) and 93% (92.6 µg/mL) of Vox were released from the pure drug and F1, respectively. Consistent with the previous study [50], micelles contained in the simulated intestinal media may increase drug solubility and dissolution.

### 3.6. In Vitro Lipolysis and X-ray Powder Diffraction of the Precipitates

**Figure 7:**
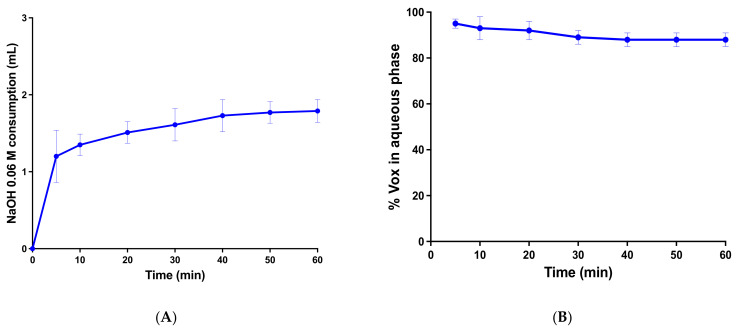
(**A**) NaOH consumption of SNEDDS during in vitro lipolysis. Data are expressed as the mean ± SD, *n* = 3. (**B**) VOX content in the aqueous phase during in vitro lipolysis. Data are expressed as the mean ± SD, *n* = 3.

**Figure 8:**
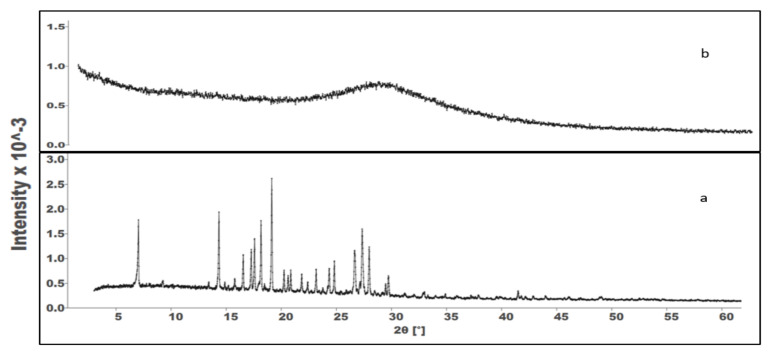
Powder X-ray diffractograms of pure Vox (**a**) and pellet-F1 (**b**).

When administered orally, SNEDDSs are prone to digestion by pancreatic lipase. The SNEDDS’s digestion in the GI tract is crucial for drug dissolution and absorption. It can be beneficial (drug solubilization) or deleterious (drug precipitation after the digestion of the oil phase). An in vitro lipolysis test was used to study the impact of GI digestion on the in vitro performance of the SNEDDSs. The test aims to reveal the ability of the SNEDDSs to maintain drug solubilization after digestion [28,51]. The consumption of NaOH during the experiment, reflecting the progress of lipolysis, is depicted in Figure 7A. The results from this study show that 88% of Vox remained in the aqueous phase after 60 min of the experiment (Figure 7B). In the aqueous phase, the drug was clearly dissolved in the mixed micelles formed by the fatty acids and monoglycerides generated during the hydrolysis of lipids [25]. To investigate the physical state of the precipitated drug after the lipolysis, a powder X-ray diffractometry of the pure drug and the resulting pellets was performed. The results in Figure 8 show that pure Vox presented peaks in the range from 5-30°, providing proof of the crystalline state of the drug. However, the pellet-F1 diffractograms showed no peaks related to crystalline Vox, suggesting that the precipitates might be in an amorphous form (or a molecular dispersed state), which could lead to rapid in vivo redissolution of the drug [52,53,54].

### 3.7. Stability of the Vox-SNEDDSs Formulation

The SNEDDS showed no physical changes during the visual observation over six months. The droplet size, zeta potential, and emulsification time of the fresh SNEDDS vs. the stored SNEDDS were 32.4 ± 0.4 vs. 31.2 ± 0.7 (s ± SD, *n* = 3), −8.4 ± 1.3 vs. −8.2 ± 0.4, and 34.9 ± 1.2 vs. 33.1 ± 2.4 (nm, SD, *n* = 3), respectively. The voxelotor content of the SNEDDS before the stability test was approximately 100%. At the end of the six months, the voxelotor content did not change significantly (>99%).

### 3.8. The Transport of Vox across Caco-2 Cell Monolayers

**Figure 9:**
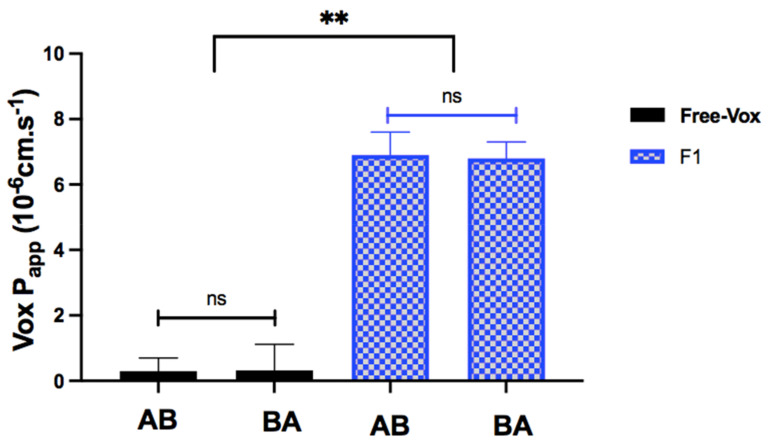
The apparent permeability (P_app_) values of Vox across the Caco-2 cell monolayer-free drug and F1 after 2 h of the AB and BA transport studies. Each value is the mean of three separate determinations. ns *p* > 0.01, ** *p* < 0.01.

To select the SNEDDS concentration to be used in the transport studies, the cytotoxicity of F1 was tested in Caco-2 cells. The Caco-2 cell viability was higher than 80% following the exposure to 0.9 mg/mL Vox suspension or unloaded F1 for up to 2 h. A 0.9 mg/mL Vox suspension was used instead of 75 µg/mL to increase the chances of drug detection on the basolateral side. Based on these results, 2-h transport studies were conducted with 0.9 mg/mL free Vox dispersed in HBSS, or Vox-F1 (corresponding to 75 µg/mL Vox). The TEER values before and after the incubation period did not change (*p* > 0.05). The AB transport of Vox from the pure drug suspension and F1 is presented in Figure 9. The amounts of Vox transported from the drug suspension and F1 were 0.062 µg and 1.4 µg, respectively. The P_app_ values of Vox from F1 were 22-times (*p* < 0.01) higher than those of the free drug. This phenomenon could be explained by the observation that the Cremophor-EL^®^ and Labrafil M^®^ 1944 CS used in the F1 formulation could contribute to the enhancement of drug solubility and permeation across Caco-2 monolayers, by opening the tight junctions and increasing the membrane fluidity [55]. To investigate the potential existence of an active efflux during drug permeation, BA transport experiments were conducted. The P_app_ values of Vox from the BA transport are shown in Figure 9 and are compared to those of the AB transport. No significant difference was observed between the P_app_ values from the AB and BA transport of the free Vox or Vox-F1, indicating that Vox was not a substrate of P-gp. These observations are in line with studies reported by Metcalf et al. [56].

### 3.9. Pharmacokinetics Study

**Figure 10:**
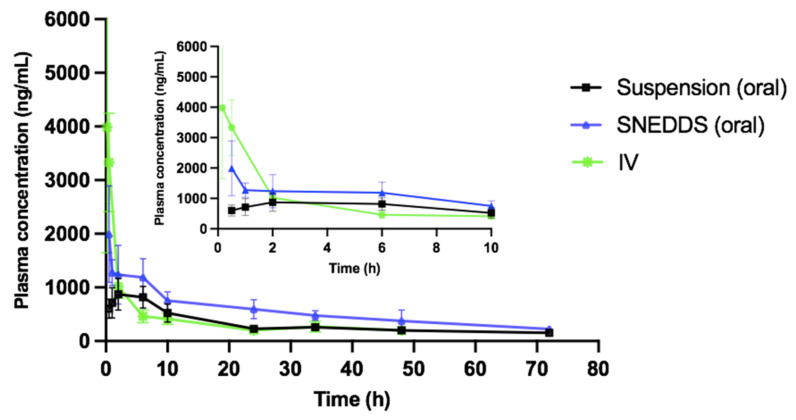
Plasma concentration time profiles of voxelotor after bolus intravenous injection (1.6 mg/kg) and oral administration of the suspension or SNEDDS (7.2 mg/kg) to rats. Data are expressed as the mean ± SD, *n* = 6.

To assess whether the SNEDDS could enhance the oral bioavailability of VOX, the pharmacokinetic parameters of the Vox suspension, the SNEDDS, and the IV solution were evaluated in rats. The plasma concentration vs. the time profile of Vox after the administration of various formulations is shown in Figure 10, and their mean pharmacokinetic data are summarized in Table 6. The AUC of Vox in F-1 increased 1.7-fold, compared to that of the drug suspension (39,469 ng∙h/mL vs. 22,530 ng∙h/mL, *p* < 0.01). The T_max_ of the drug suspension and F-1 was found to be 2 h and 0.5 h, respectively. This result indicates a rapid absorption of Vox from the formulation. The C_max_ of the optimized formulation (1994 ng/mL) and the drug suspension (874 ng/mL) exhibited a nearly 2.3-fold enhancement (*p* < 0.01). Finally, the absolute oral bioavailability of Vox from the SNEDDS resulted in a 1.7-fold increase, compared to the drug in suspension. This increased bioavailability might be due to the improved drug solubility, the synergistic effect of the surfactant and oil as absorption enhancers, and the avoidance of the first pass metabolism via the lymphatic transport.

## 4. Conclusions

In this study, the Qbd approach was applied to develop Vox-SNEDDSs with improved aqueous solubility and oral bioavailability. Solubility and emulsification studies suggested the suitability of Capryol PGMC^®^, Cremophor-EL^®^, and Labrafil M^®^ 1944 as oils, surfactants, and cosurfactants, respectively. Ternary diagram studies indicated the nanoemulsification region and range of factors that should be applied in the DoE. The D-optimal design suggested that the SNEDDSs’ formulation comprised of Capryol PGMC^®^ (40% *w*/*w*), Cremophor-EL^®^ (43% *w*/*w*), and Labrafil M^®^ 1944 CS (17% *w*/*w*). Thus, it fulfilled the maximum requirements of an optimum formulation and was chosen for further evaluation. The optimized formulation showed an emulsification time of 32.4 ± 0.4 s, globule size of 34.9 ± 1.2 nm, polydispersity index of 0.204 ± 0.0, zeta potential of −8.4 ± 1.3 mV, and transmittance percentage of 99.2 ± 0.6%. The spherical shape of the oil globules in the nanoemulsion was revealed using transmission electron microscopy. The optimized SNEDDS revealed a high drug dissolution, and at least 88% of the Vox remained solubilized after the in vitro lipolysis of the formulation. The in vitro transport study across Caco-2 cell monolayers revealed that the SNEDDSs could significantly enhance the permeation of Vox compared to the free drug. Thus, the developed SNEDDS resulted in a 1.7-fold higher oral bioavailability of Vox in rats, compared to the drug suspension. This new SNEDDS may be further developed as an alternative formulation of voxelotor.

## Figures and Tables

**Table 1:** Ability of the selected surfactants to emulsify the oil Capryol PGMC^®^.

Surfactant	% Transmittance
Labrasol ALF^®^	60.5 ± 0.86 *
Tween^®^ 80	95.1 ± 0.79
Cremophor-EL^®^	99.3 ± 0.69

* *p* < 0.05 compared to Cremophor 80.

**Table 2:** D-optimal mixture design and response data for the optimization of SNEDDSs.

	Oil (mg)	Surfactant (mg)	Cosurfactant (mg)	Y_1_ (s)	Y_2_ (nm)	Y_3_	Y_4_ (%)
1	295	605	100	82	47	0.57	98
2	200	560	240	57	27	0.16	100
3	400	300	300	13	60	0.15	88
4	475	375	150	15	48	0.18	97
5	600	300	100	10	83	0.24	80
6	200	700	100	113	45	0.78	97
7	460	300	240	12	65	0.19	83
8	310	545	145	47	30	0.26	97
9	520	300	180	9.6	69	0.22	86
10	280	420	300	65	39	0.04	97
11	480	420	100	46	35	0.08	99
12	200	640	160	11	32	0.40	99
13	400	300	300	11	60	0.17	92
14	200	500	300	94	28	0.05	100
15	330	430	240	54	32	0.03	99
16	600	300	100	10	86	0.24	82

Y_1_: emulsification time, Y_2_: droplet size, Y_3_: PDI, Y_4_: transmittance percentage.

**Table 3:** Model fitting and statistical analysis.

Reponses	Model	R-Square	Adjusted R-Square	Root Mean Square Error	Prob ≥ F
Y_1_	Quadratic	0.945	0.917	11.76	0.0036 *
Y_2_	Cubic	0.985	0.964	3.614	0.0001 *
Y_3_	Quadratic	0.907	0.861	0.074	0.0001 *
Y_4_	Cubic	0.985	0.963	1.67	0.0018 *

* Significant.

**Table 4:** Summary of ANOVA for the response parameters.

	Term	Estimate	Std Error	DFDen	t Ratio	Prob>|t|
**Y_1_**	(X_1_ − 0.2)/0.4	11.92	8.82	9.973	1.35	0.2065
	(X_2_ − 0.3)/0.4	114.22	11.15	9.942	10.24	<0.0001 *
	(X_3_ − 0.1)/0.4	144.92	65.71	7.93	2.21	0.0588
	X_1_ × X_2_	−26.484	45.33	7.804	−0.58	0.5756
	X_1_ × X_3_	−230.63	123.98	7.791	−1.86	0.1009
	X_2_ × X_3_	−189.74	120.94	7.461	−1.57	0.1580
**Y_2_**	(X_1_ − 0.2)/0.4	84.62	2.87	5.211	29.44	<0.0001 *
	(X_2_ − 0.3)/0.4	46.44	4.00	4.867	11.61	<0.0001 *
	(X_3_ − 0.1)/0.4	71.56	122.67	3.808	0.58	0.5924
	X_1_ × X_2_	−125.70	19.65	4.75	−6.40	0.0017*
	X_1_ × X_3_	−70.86	243.76	3.812	−0.29	0.7864
	X_2_ × X_3_	−117.35	241.13	3.79	−0.49	0.6533
	X_1_ × X_2_ × X_3_	23.12	336.44	3.837	0.07	0.9486
	X_1_× X_2_ × (X_1_ − X_2_)	−114.32	29.27	3.975	−3.91	0.0177 *
	X_1_ × X_3_ × (X_1_ − X_3_)	6.80	164.65	3.903	0.04	0.9691
	X_2_ × X_3_ × (X_2_ − X_3_)	−46.16	160.425	3.887	−0.29	0.7882
**Y_3_**	(X_1_ − 0.2)/0.4	0.139	0.052	9.759	2.65	0.0248 *
	(X_2_ − 0.3)/0.4	0.792	0.067	9.948	11.75	<0.0001 *
	(X_3_ − 0.1)/0.4	0.084	0.421	8.384	0.20	0.8464
	X_1_ × X_2_	−0.844	0.3004	8.609	−2.81	0.0213*
	X_1_ × X_3_	0.272	0.794	8.16	0.34	0.7407
	X_2_ × X_3_	−1.735	0.776	7.364	−2.24	0.0586
**Y_4_**	(X_1_ − 0.2)/0.4	80.88	1.46	5.673	55.33	<0.0001 *
	(X_2_ − 0.3)/0.4	97.97	1.93	4.726	50.81	<0.0001 *
	(X_3_ − 0.1)/0.4	151.52	55.02	3.025	2.75	0.0698
	X_1_ × X_2_	43.26	8.60	3.256	5.03	0.0124*
	X_1_ × X_3_	−107.66	109.18	3.023	−0.99	0.3963
	X_2_ × X_3_	−102.28	108.09	3.018	−0.95	0.4135
	X_1_ × X_2_ × X_3_	161.85	149.42	3.018	1.08	0.3576
	X_1_ × X_2_ × (X_1_ − X_2_)	49.33	13.03	3.065	3.79	0.0311 *
	X_1_ × X_3_ × (X_1_ − X_3_)	74.99	73.51	3.047	1.02	0.3817
	X_2_ × X_3_ × (X_2_ − X_3_)	77.48	71.94	3.049	1.08	0.3592

* Significant.

**Table 5:** The predicted values and experimental results of F1 prepared under optimum conditions.

Response	Predicted Value	Experimental Value	Bias (%)
Y_1,_ emulsification time (s)	33.1	32.4 ± 0.4	2.1
Y_2,_ droplet size (nm)	33.8	34.9 ± 1.2	−3.2
Y_3,_ PDI	0.210	0.20 ± 0.0	2.9
Y_4_, transmittance (%)	99.4	99.2 ± 0.6	0.2
Zeta potential (mV)	--	−8.4 ± 1.3	--

Bias (%) = (predicted value − observed value)/observed value × 100. --: not determined.

**Table 6:** Pharmacokinetic parameters.

Parameters	Suspension	SNEDDS	IV
Dose (mg/kg)	7.2	7.2	1.6
T_1/2_ (h)	50.5	32.6	24.6
T_max_ (h)	2	0.5	--
C_max_ (ng/mL)	873.8 ± 294.4	1993.9 ± 892.5 **	3984.5 ± 239.5
AUC_0–72_ (h.ng/mL)	22,529.7 ± 146.1	39,468.9 ± 580.2 **	19,748.0 ± 420
F (%)	25.4	44.4	--

Each value is the mean ± SEM of six rats. ** *p* < 0.01 compared to Vox suspension.

## Data Availability

The data presented in this study are available on request from the corresponding author.

## Supplementary Materials

The following are available online at https://www.mdpi.com/article/10.3390/pharmaceutics13091388/s1, Table S1. Validation results obtained for the HPLC quantification method of voxelotor, Table S2. Emulsification study of surfactant/cosurfactant combinations, Figure S1. Accuracy profile of HPLC mathod obtained with four concentration levels of voxelotor. The plain line is the relative bias, dashed lines are the β-expectation tolerance limits (β = 95%) and dotted lines represent the acceptance limits (± 20%). The dots represent the relative back-calculated concentrations of the validation standards and are plotted according to their target concentration, Figure S2. Linear profile of HPLC method obtained with four concentration levels of Voxelotor. The plain line is the identity line (y = x), the dashed line is the β-expectation tolerance limits (β = 95%) and dotted lines represent the acceptance limits (±20%).
