# Supplementary material for: Quality-by-Design-Based Development of a Voxelotor Self-Nanoemulsifying Drug-Delivery System with Improved Biopharmaceutical Attributes
Source: Pharmaceutics. 2021 Sep 2;13(9):1388. doi: 10.3390/pharmaceutics13091388 (PMC8468394; doi:10.3390/pharmaceutics13091388)
Supplement: Supplementary file 1 [file pharmaceutics-13-01388-s001.zip › pharmaceutics-1320121-supplementary.pdf]

pharmaceutics

Article

Quality-by-design-based development of a voxelotor self-nanoemulsifying drug-delivery system with improved biopharmaceutical attributes

Aristote B. Buya[1,3], Romano Terrasi[2], Jérémie K. Mbinze[3], Giulio G. Muccioli[2], Ana Beloqui[1], Patrick B. Memvanga[3], Véronique Préat[1*]

Supplementary materials

Table S1. Validation results obtained for the HPLC quantification method of voxelotor.

Validation criteria		Concentration levels (µg/mL) for voxelotor			
		2.5	10	25	75
Response function					
Trueness	Relative bias (%)	0.0018	-4.0894	1.5739	5.8087
Precision	Repeatability (RSD %)	0.5863	1.2562	0.6054	3.0666
	Intermediate precision (RSD %)	0.9519	2.7611	1.9830	2.5451
Accuracy (95% relative β-expectation lower and		-3.3838	-14.0593	-8.2013	-0.4523
Upper tolerance limits in %)		3.3875	5.8804	11.3492	12.0697
Uncertainty (µg/mL)		0.0265	0.3000	0.5769	2.0305
Expanded uncertainty (µg/mL)		0.053	0.0531	1.1538	4.0610
Relative expanded uncertainty (%)		2.1277	6.0093	4.6153	5.4147
Linearity	slope	1.0659			
	Intercept	-0.7684			
	R^2	0.9986			

Table S2. Emulsification study of surfactant/cosurfactant combinations.

Cosurfactant	% Transmittance
	Cremophor-EL®
Transcutol HP®	99.8 ± 0.85
Propylene glycol	99.4 ± 0.45
Polyethylene glycol 400	99.6 ± 1.62
Labrafil M® 1944 CS	98.3 ± 0.65
Labrafil M® 2125 CS	89.4 ± 0.15

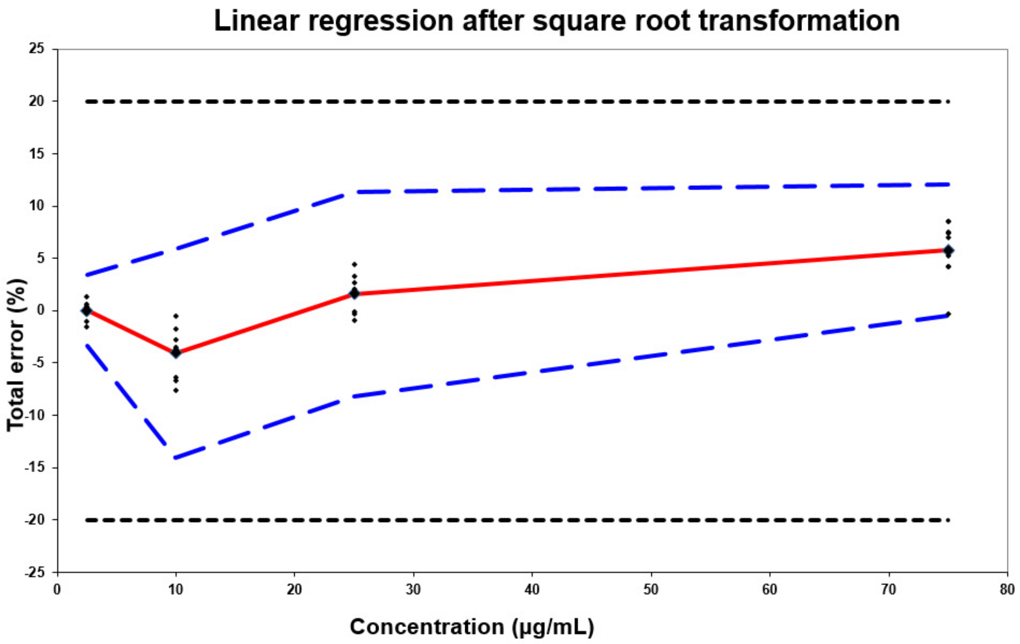

Figure S1. Accuracy profile of HPLC mathod obtained with four concentration levels of voxelotor. The plain line is the relative bias, dashed lines are the β-expectation tolerance limits (β=95%) and dotted lines represent the acceptance limits (±20%). The dots represent the relative back-calculated concentrations of the validation standards and are plotted according to their target concentration.

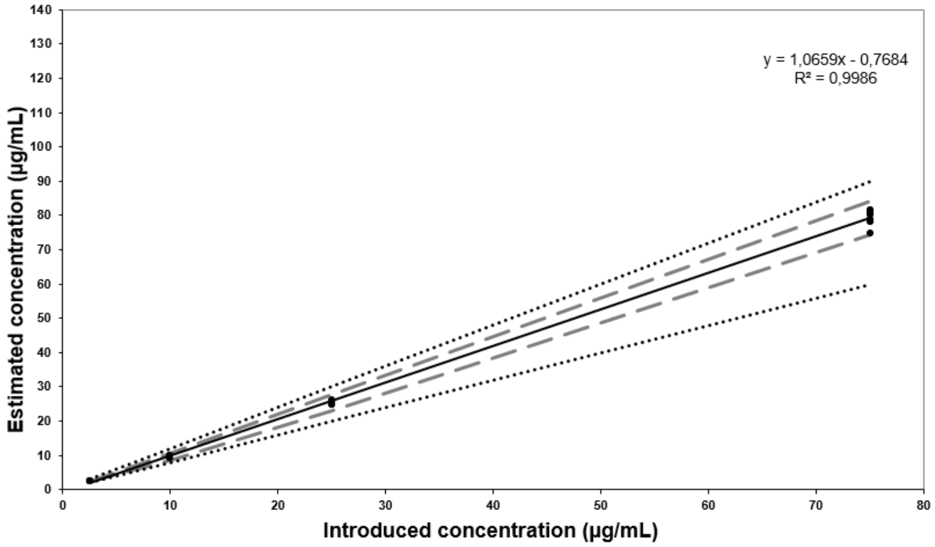

Figure S2. Linear profile of HPLC method obtained with four concentration levels of Voxelotor. The plain line is the identity line (y=x), the dashed line is the β-expectation tolerance limits (β=95%) and dotted lines represent the acceptance limits (±20%).
